# β-arrestin-2 enhances intestinal epithelial apoptosis in necrotizing enterocolitis

**DOI:** 10.18632/aging.102320

**Published:** 2019-10-14

**Authors:** Dong Fu, Peng Li, Qingfeng Sheng, Zhibao Lv

**Affiliations:** 1 Department of General Surgery, Children’s Hospital of Shanghai, School of Medicine, Shanghai Jiao Tong University, Shanghai 200000, China

**Keywords:** β-arrestin-2, NEC, BiP, ER stress, apoptosis

## Abstract

Apoptosis among intestinal epithelial cells contributes to necrotizing enterocolitis (NEC), a severe intestinal disease that particularly affects premature infants. β-arrestin-2, an important regulator of G-protein-coupled receptors, is expressed in intestinal epithelial cells, where its activation promotes apoptosis. We found that β-arrestin-2 was overexpressed in both human and murine NEC samples. β-arrestin-2-deficient mice were protected from endoplasmic reticulum stress and NEC development. The endoplasmic reticulum-resident chaperone BiP was found to promote intestinal epithelial cell survival. Pretreatment of intestinal epithelial cells or mice with the BiP inhibitor HA15 increased cell apoptosis and promoted NEC development. β-arrestin-2 bound to BiP and promoted its polyubiquitination and degradation, thereby facilitating the release of the pro-apoptotic molecule BIK from BiP. Silencing β-arrestin-2 downregulated apoptosis by increasing BiP levels, which suppressed endoplasmic reticulum stress. This study suggests that β-arrestin-2 induces NEC development by inhibiting BiP, thereby triggering apoptosis in response to endoplasmic reticulum stress. Thus, novel therapeutic strategies to inhibit β-arrestin-2 may enhance the treatment of NEC.

## INTRODUCTION

Necrotizing enterocolitis (NEC) is one of the most common gastrointestinal emergencies in premature infants, and is associated with significant morbidity and mortality [1]. Although factors such as prematurity, hypoxia, enteral feeding and bacterial colonization have been implicated in the pathogenesis of NEC, the precise mechanism of its development remains to be delineated.

Endoplasmic reticulum (ER) stress-induced apoptosis of intestinal epithelial cells (IECs) has come to be recognized as an important promoter of most gastrointestinal diseases [2]. IECs are highly secretory cells in the gastrointestinal tract. As secretion and protein folding are both extremely energy-dependent processes, they compete with each other for cellular energy. Thus, IECs are quite prone to incorrect protein folding, and the gastrointestinal tract is more susceptible to ER stress than other organs [3].

The unfolded protein response is a stress response that allows cells to cope with ER stress [4]. The genetic knockdown of proteins involved in the unfolded protein response has been reported to cause Paneth cell dysfunction or apoptosis, and to increase the susceptibility of mice to dextran sodium sulfate (DSS)-induced colitis and spontaneous intestinal inflammation [5]. Binding immunoglobulin protein/glucose-regulated protein 78 (BiP/GRP78) is an important molecular chaperone that maintains physiological homeostasis by sensing and promoting the unfolded protein response under ER stress [6, 7]. In addition, BiP can inhibit apoptosis by directly binding and suppressing the release of caspase 7, caspase 12 or BIK [8–10]. BiP itself is downregulated by polyubiquitination and proteasomal degradation [11].

β-arrestin-2, a ubiquitously expressed cytosolic protein, is well-characterized as an adaptor protein that facilitates endocytosis and attenuates seven-transmembrane receptor signaling [12]. In addition to promoting receptor desensitization and internalization, β-arrestin-2 functions as a scaffold protein and a signaling molecule in G-protein-coupled receptor signaling pathways, such as those involving mitogen-activated protein kinases, extracellular signal-regulated kinase 1/2 (ERK1/2), c-Jun N-terminal kinases, p38 kinases, Src family kinases and E3 ligase MDM2 [12–15]. β-arrestin-2 can bind to various proteins and alter their phosphorylation, ubiquitination and/or subcellular distribution. Thus, β-arrestin-2 regulates a wide variety of cellular responses, including proliferation, apoptosis, chemotaxis, immunity, gene transcription and protein translation [16]. BiP has been reported to bind directly to β-arrestin-2 [17].

We hypothesized that β-arrestin-2 induces pathological ER stress by binding to BiP, thus increasing BIK release, stimulating apoptosis and promoting the development of NEC. The susceptibility of premature infants to NEC may be partly ascribed to their higher baseline intestinal β-arrestin-2 expression.

## RESULTS

### β-arrestin-2 promotes NEC in humans, mice and IECs

**Figure 1:**
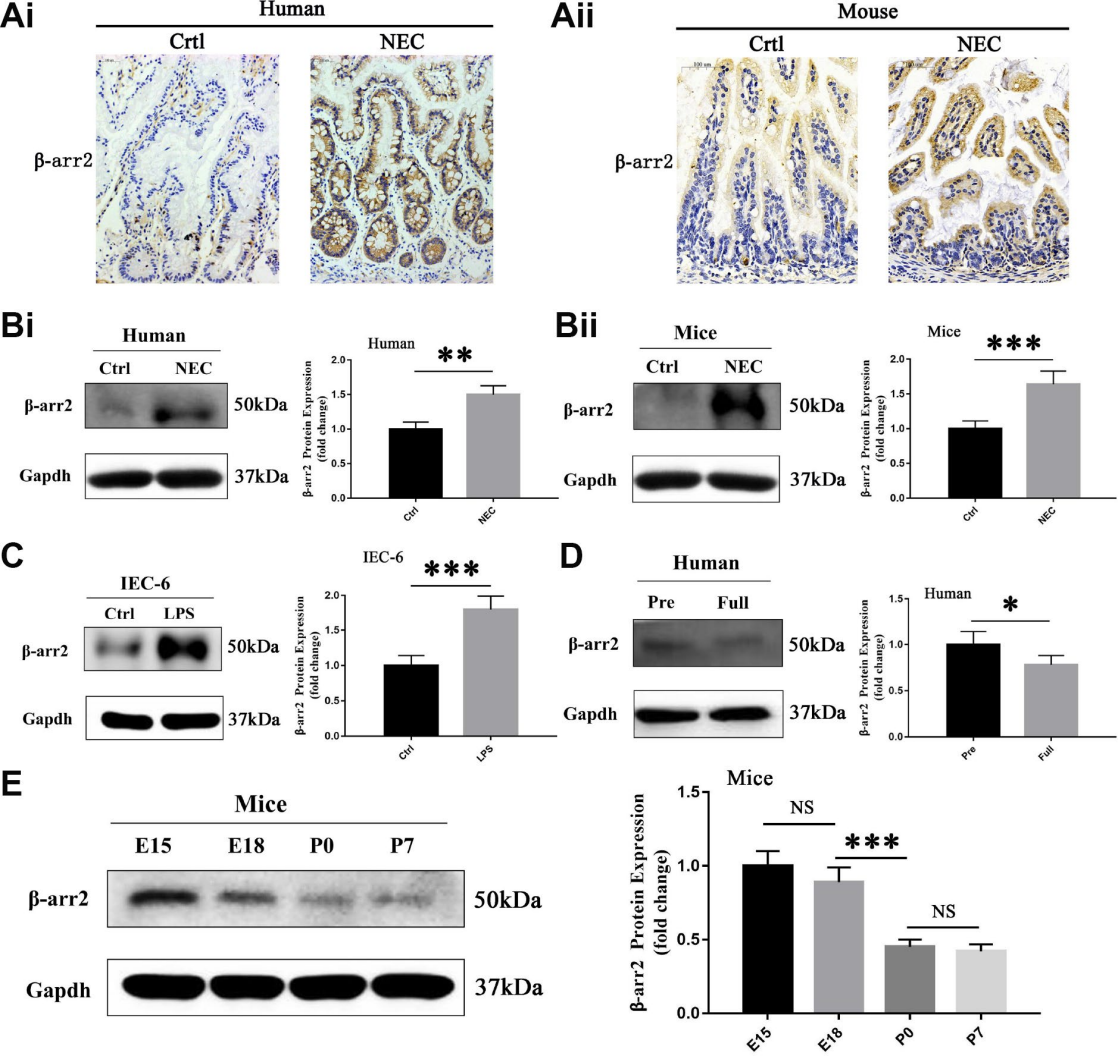
**β-arrestin-2 is upregulated in NEC in humans, mice and IECs.** (**A**) Immunohistochemical staining demonstrated that β-arrestin-2 expression was greater in both humans and mice with NEC than in healthy controls. (**B**) β-arrestin-2 protein was detected in the neonatal intestine, and was upregulated in NEC samples from both humans (n=6) and mice (n=6). (**C**) LPS stimulation increased β-arrestin-2 expression in IEC-6 cells. (**D**) β-arrestin-2 expression was greater in premature than in full-term human infants (n=6). (**E**) In mice, β-arrestin-2 levels in the distal ileum trended down from embryonic day 15 (E15) to postnatal day 7 (P7) (n=6).

To investigate the potential involvement of β-arrestin-2 in the pathogenesis of NEC, we first analyzed β-arrestin-2 expression in neonatal intestinal tissues from humans and mice. The demographic and clinical characteristics of the NEC patients and control patients are shown in Supplementary Table 1. Immunohistochemical staining revealed that β-arrestin-2 was expressed in neonatal intestinal tissues, and was upregulated in both humans (Figure 1Ai) and mice (Figure 1Aii) with NEC compared with healthy controls. Western blotting also revealed that β-arrestin-2 protein expression was upregulated in intestinal samples from humans (Figure 1Bi) and mice (Figure 1Bii) with NEC (P < 0.05). Furthermore, β-arrestin-2 expression was markedly greater in lipopolysaccharide (LPS)-treated IEC-6 cells than in control cells (Figure 1C).

As preterm infants are vulnerable to NEC, we measured the expression of β-arrestin-2 at different time points during intestinal development. β-arrestin-2 expression was lower in full-term than in preterm infants (P < 0.05) (Figure 1D). In mice, β-arrestin-2 expression in the distal ileum at P0 (full-term day 0) was significantly lower than that at E18 (embryonic day 18), but did not differ significantly from that at P7 (full-term day 7) (Figure 1E). These results suggested that β-arrestin-2 expression may be associated with the occurrence of NEC during the neonatal stage.

### Targeted deletion of β-arrestin-2 impedes the development of NEC

**Figure 2:**
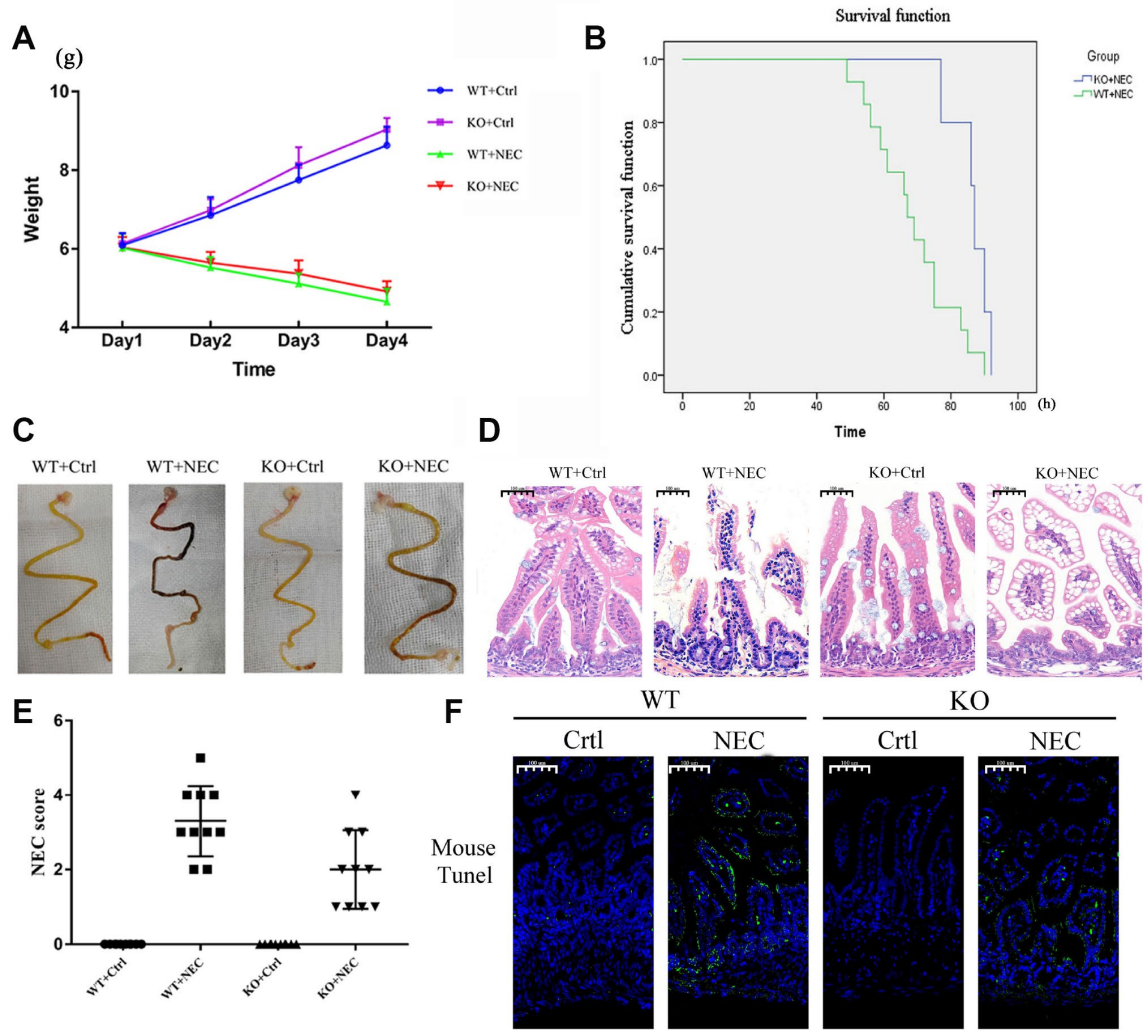
**Targeted deletion of β-arrestin-2 impeded the development of NEC.** (**A**) The body weights of mice decreased more slowly in the KO+NEC group (red) than in the WT+NEC group (green) (n=20). (**B**) The survival rate was much higher in the KO+NEC group (blue) than in the WT+NEC group (green) (n=20). (**C**) Representative images of gross morphology demonstrate that edema, congestion, necrosis and reddish-black coloring were more severe in the WT+NEC group than in the KO+NEC group. (**D**) Representative images display the more serious histological changes in the WT+NEC group, including the shedding of epithelial cells, necrosis of the entire villus and transmural necrosis. (**E**) The NEC score was lower in the KO+NEC group (2.00±1.05) than in the WT+NEC group (3.30±0.95) (P=0.01) (n=20). (**F**) A TUNEL assay revealed a much lower proportion of apoptotic cells in the KO+NEC group than in the WT+NEC group.

To determine whether β-arrestin-2 promotes the development of NEC, we experimentally induced NEC in β-arrestin-2 knockout (KO) and wide-type (WT) mice, and compared the mice with the corresponding controls (Ctrl). Body weights were recorded for all four groups (WT+Ctrl, WT+NEC, KO+Ctrl, KO+NEC), and although both of the NEC groups lost weight, the body weight decline was slower in the KO+NEC group than in the WT+NEC group (Figure 2A). The survival rates of the NEC groups also were examined. Death events began on experimental day 3 in the WT+NEC group and continued to occur rapidly thereafter. By the end point, only 30% of mice in the WT+NEC group had survived, while 75% of mice in the KO+NEC group had survived (Figure 2B). Thus, significantly better survival was observed in the KO+NEC group.

Regarding the gross morphology of the intestine, more severe changes were observed in the WT+NEC group than in the KO+NEC group, including edema, congestion, necrosis and a reddish-black coloring (Figure 2C). In terms of histological changes, the shedding of epithelial cells, necrosis of the entire villus and transmural necrosis were more serious in WT+NEC mice than in KO+NEC mice (Figure 2D). The NEC score was also significantly lower in β-arrestin-2 knockout mice than in wild-type mice (WT+NEC 3.30 ± 0.95 vs. KO+NEC 2.00 ± 1.05, P = 0.01) (Figure 2E). Immunofluorescence revealed that TUNEL staining was lower in the KO+NEC group than in the WT+NEC group (Figure 2F). These results further confirmed that β-arrestin-2 is critical for the development of NEC.

In the animal model, it was intriguing to note that when we inserted the disposable silicone catheter (19Fr for newborns) into the stomachs of the mice, the knockout mice held the tube tightly and began sucking on it, resembling hungry human infants. However, the wild-type mice desperately struggled and rejected the tube (Supplementary Figure 1). Thus, we speculated that the deletion of β-arrestin-2 in this NEC mouse model affected not only the gastrointestinal tract, but also the central nervous system, enabling the pups to adapt to their environment with greater intelligence.

### β-arrestin-2 promotes the development of NEC by inducing ER-stress-associated apoptosis

**Figure 3:**
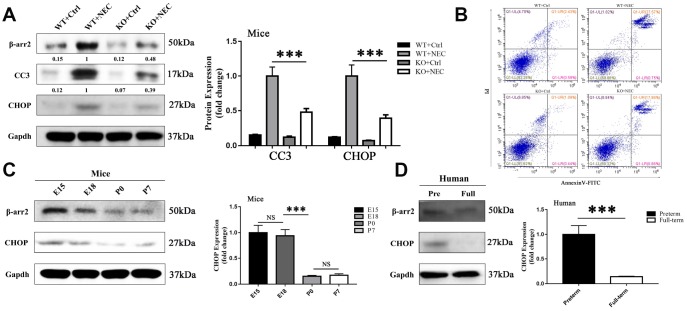
**β-arrestin-2 promotes the development of NEC by inducing ER stress.** (**A**) Cleaved caspase 3 (CC3) expression in KO+NEC mice was less than half of that in WT+NEC mice (n=5). (**B**) Flow cytometry analysis revealed a greater proportion of apoptotic IECs in WT+NEC mice (27.57%) than in KO+NEC mice (17.98%) (n=5). (**C**) In mice, CHOP expression trended down from E15 to P7 (n=3). (**D**) In humans, CHOP expression was greater in premature than in full-term infants (n=5).

To investigate whether ER-stress-associated apoptosis was involved in β-arrestin-2-induced NEC development, we measured apoptosis and ER stress levels in the intestines of the WT+Ctrl, WT+NEC, KO+Ctrl and KO+NEC groups. Apoptosis was evaluated based on the expression of cleaved caspase 3 (CC3). In both β-arrestin-2 knockout mice and wild-type mice, CC3 expression was greater in the NEC group than in the Ctrl group; however, in the KO+NEC group, the increase in CC3 expression was less than half of that in the WT+NEC group (Figure 3A). In addition, apoptosis was measured by flow cytometry with Annexin V/propidium iodide (PI) staining in IECs isolated from the four groups. The proportion of apoptotic IECs in the WT+NEC group (28.32%) was more than 50% greater than that in the KO+NEC group (18.84%) (Figure 3B).

The mechanism of intestinal mucosal cell apoptosis in NEC is still poorly understood, but ER stress may be a leading contributor. The expression of CCAAT-enhancer-binding protein homologous protein (CHOP), a transcription factor in the ER stress response, was upregulated in both NEC groups. However, like CC3, CHOP was not equally induced in the two NEC groups; CHOP expression was 2.5-fold greater in the WT+NEC group than in the KO+NEC group (Figure 3A). Furthermore, CHOP expression was greater in preterm mice (Figure 3C) and infants (Figure 3D) than in full-term ones. Hence, β-arrestin-2 expression appeared to correlate with CHOP and CC3 expression. These results suggested that β-arrestin-2 may induce NEC by stimulating ER-stress-induced apoptosis.

### β-arrestin-2 promotes ER-stress-associated apoptosis by suppressing BiP

**Figure 4:**
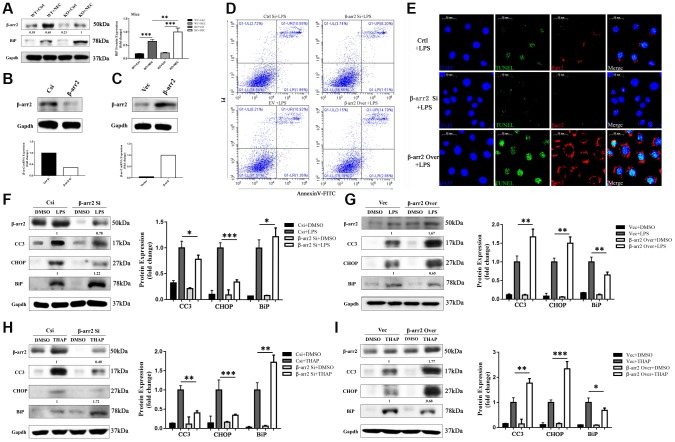
**β-arrestin-2 promotes ER stress by suppressing BiP.** (**A**) BiP was upregulated by more than 50% in the KO+NEC group compared with the WT+NEC group. (**B**) Specific siRNA reduced β-arrestin-2 expression, as determined by real-time PCR and Western blotting. (**C**) Plasmid transfection efficiently increased β-arrestin-2 expression, as determined by real-time PCR and Western blotting. (**D**) Flow cytometry analysis revealed that apoptosis increased when β-arrestin-2 was overexpressed and decreased when β-arrestin-2 was silenced. (**E**) A TUNEL assay revealed that apoptosis was attenuated when β-arrestin-2 was silenced. (**F**) Compared with control siRNA (Csi), siRNA against *β-arrestin-2* reduced CC3 expression by 22% and increased BiP expression by 22% in LPS-treated cells. (**G**) Compared with the control vector (Vec), overexpression of β-arrestin-2 increased CC3 expression by 67% and reduced BiP expression by 35% in LPS-treated cells. (**H**, **I**) As a positive control, thapsigargin (THAP) yielded similar results to LPS.

BiP has been reported to be an important regulator of ER stress and protector of cell survival. In our study, BiP expression was greater in both NEC groups than in their corresponding control groups. Like CC3 and CHOP, BiP was unequally upregulated in the two NEC groups; however, in contrast to the former two proteins, BiP was upregulated to a significantly greater extent in the KO+NEC group than in the WT+NEC group (Figure 4A). Thus, β-arrestin-2 expression appeared to correlate negatively with BiP expression.

We next used small interfering RNA (siRNA) to silence β-arrestin-2 in IEC-6 cells. Real-time PCR analysis revealed that the mRNA expression of *β-arrestin-2* was reduced two- to three-fold by the siRNA treatment, and Western blot analysis confirmed that β-arrestin-2 protein expression was also reduced (Figure 4B). On the other hand, plasmid transfection efficiently overexpressed β-arrestin-2 (Figure 4C). Flow cytometry analysis of LPS-treated cells demonstrated that apoptosis increased when β-arrestin-2 was overexpressed and decreased when β-arrestin-2 was silenced (Figure 4D). A TUNEL assay further confirmed that silencing β-arrestin-2 markedly attenuated LPS-induced apoptosis (Figure 4E).

LPS treatment induced the expression of the apoptotic marker CC3 and the ER stress markers CHOP and BiP in IEC-6 cells (Figure 4F, 4G). Treatment with β-arrestin-2 siRNA attenuated the LPS-induced increases in CC3 and CHOP expression, but augmented the increase in BiP expression (Figure 4F). On the other hand, overexpression of β-arrestin-2 enhanced the LPS-induced increases in CC3 and CHOP expression, but dampened the increase in BiP expression (Figure 4G). Similar results were observed when the same cell lines were pretreated with thapsigargin (a positive control for ER stress) rather than LPS (Figure 4H, 4I). These results suggested that BiP is an important downstream protein that links β-arrestin-2 with ER-stress-associated apoptosis.

### BiP suppresses β-arrestin-2-induced pro-apoptotic effects

**Figure 5:**
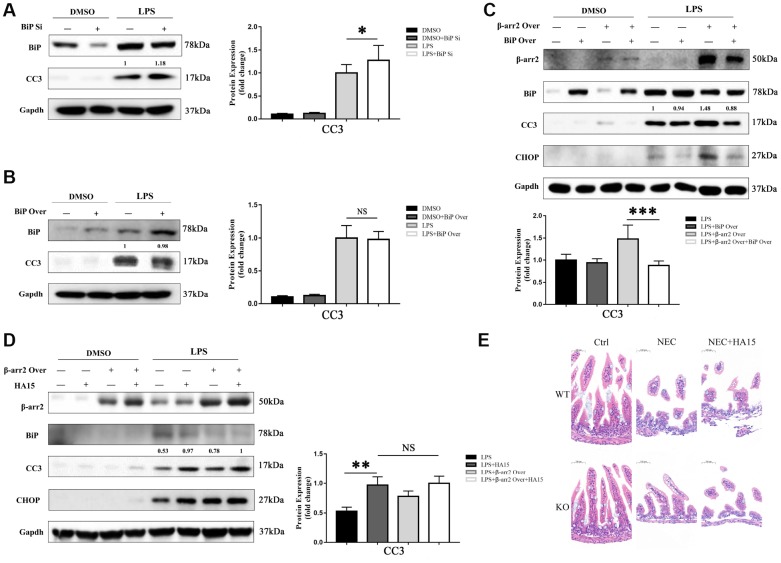
**BiP suppresses β-arrestin-2-induced pro-apoptotic effects.** (**A**) Silencing of BiP increased apoptotic marker expression in LPS-stimulated cells. (**B**) Overexpression of BiP did not significantly alter apoptotic marker expression in LPS-stimulated cells. (**C**) Co-transfection of *β-arrestin-2* and *BiP* significantly inhibited apoptosis compared with *β-arrestin-2* transfection alone in LPS-treated cells (P<0.05). (**D**) HA15 inhibited BiP protein expression and increased CC3 expression in LPS-treated cells, regardless of whether or not β-arrestin-2 was overexpressed. (**E**) KO+NEC and WT+NEC mice pretreated with HA15 displayed almost the same degree of intestinal damage.

We next evaluated the effects of knocking down or overexpressing BiP in IECs. The knockdown or upregulation of BiP did not significantly alter the extent of apoptosis in dimethyl sulfoxide (DMSO)-treated cells. However, in LPS-treated cells, BiP knockdown significantly increased the expression of the apoptotic marker CC3, although BiP overexpression did not significantly reduce CC3 expression compared with empty vector treatment (Figure 5A, 5B).

Next, a stable *β-arrestin-2* and *BiP* co-transfectant was established. In DMSO-treated cells, no significant difference in apoptotic marker expression was detected among the groups with and without the overexpression vectors (alone or together). However, following pretreatment with LPS, cells co-transfected with *BiP* and *β-arrestin-2* exhibited significantly lower CC3 expression than those transfected with *β-arrestin-2* alone (Figure 5C). These results suggested that BiP protected IECs against β-arrestin-2-induced pro-apoptotic effects during ER stress, but only when β-arrestin-2 was overexpressed.

For further investigation, we pretreated IEC-6 cells with thiazole benzenesulfonamide compound HA15 (a novel inhibitor of BiP) [18] to block BiP synthesis before stimulating the cells with LPS. HA15 did not alter the expression of BiP, CHOP or CC3 in DMSO-treated cells, indicating that it had no pro-apoptotic effects under normal conditions; however, HA15 strongly inhibited BiP expression and increased CC3 levels in LPS-treated cells (Figure 5D). Another interesting finding was that HA15-induced apoptosis was maintained at a high level in LPS-treated cells, regardless of whether or not β-arrestin-2 was overexpressed (Figure 5D). Therefore, it is likely that β-arrestin-2 induces IEC apoptosis mainly by downregulating BiP expression. In our animal models, KO+NEC and WT+NEC mice pretreated with HA15 exhibited almost the same degree of intestinal damage (Figure 5E), further confirming the involvement of BiP in β-arrestin-2-induced ER stress and apoptotic signaling.

### β-arrestin-2 binds to BiP and promotes its polyubiquitination and degradation

**Figure 6:**
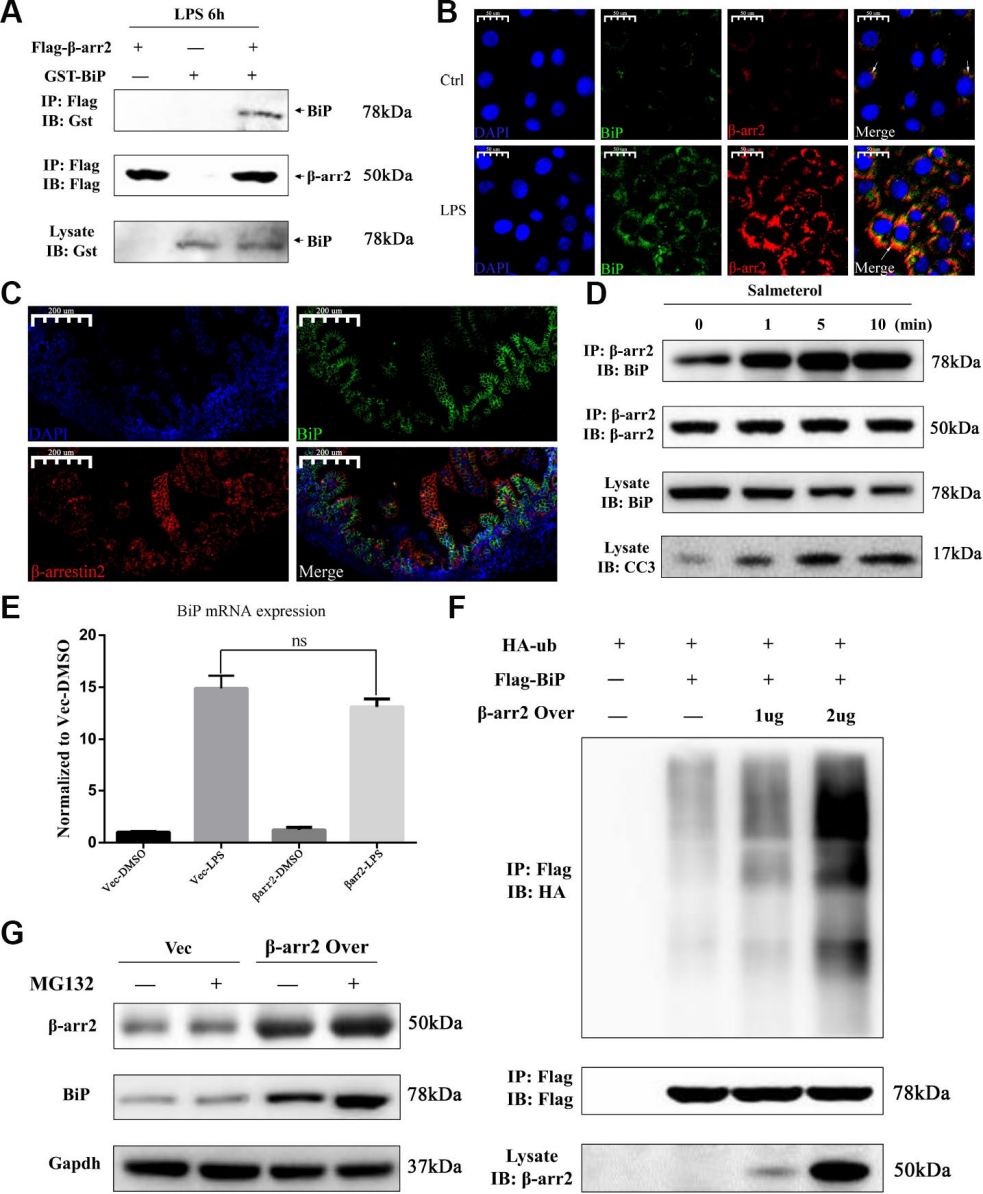
**β-arrestin-2 binds to BiP and promotes its polyubiquitination and degradation.** (**A**) A co-immunoprecipitation assay revealed that β-arrestin-2 bound directly to BiP in LPS-stimulated cells. (**B**) An immunofluorescence assay demonstrated the colocalization of β-arrestin-2 and BiP in IEC-6 cells. (**C**) An immunofluorescence assay identified cells that were double-positive for β-arrestin-2 and BiP in NEC mouse intestinal specimens. (**D**) Salmeterol time-dependently improved the binding between β-arrestin-2 and BiP, and also increased apoptotic marker expression. (**E**) *BiP* mRNA expression was not significantly influenced by β-arrestin-2 overexpression. (**F**) β-arrestin-2 increased the ubiquitination of BiP in a dose-dependent manner in LPS-stimulated cells. (**G**) The ubiquitin-proteasome inhibitor MG132 increased BiP protein levels when β-arrestin-2 was overexpressed.

We then performed a bioinformatic analysis with Gene-Mania (a biological network integration tool for gene prioritization and functional prediction), which predicted the direct physical binding between BiP and β-arrestin-2 (Supplementary Figure 2). To investigate whether the pro-apoptotic function of β-arrestin-2 was triggered by its binding to BiP in NEC, we further assessed the binding between β-arrestin-2 and BiP by co-immunoprecipitation and immunofluorescence colocalization analyses. For this purpose, we exogenously expressed β-arrestin-2 and BiP in IEC-6 cells. The co-immunoprecipitation analysis demonstrated that β-arrestin-2 directly bound to BiP in cells stimulated with LPS (Figure 6A). We next used immunofluorescence to evaluate the localization of β-arrestin-2 and BiP in IEC-6 cells, and observed that these proteins colocalized in the cytoplasm (Figure 6B). We also performed double staining for β-arrestin-2 and BiP in intestinal specimens from our NEC animal model, and detected double-positive cells (Figure 6C).

Previously, a β2-adrenergic receptor (β2AR) agonist was reported to enhance the binding of β-arrestin-2 to activated c-Src [19]. Therefore, we also investigated the binding between β-arrestin-2 and BiP in cells treated with a β2AR agonist. IEC-6 cells that co-expressed β-arrestin-2, BiP and β2AR were stimulated with the β2AR-specific agonist salmeterol at a concentration of 10^-7^ M, and were then treated with LPS. Co-immunoprecipitation analysis revealed a time-dependent improvement in the binding between β-arrestin-2 and BiP after salmeterol treatment (Figure 6D). The enhanced binding induced by salmeterol also increased apoptotic marker expression (Figure 6D).

Intriguingly, in LPS-stimulated cells, we found that the overexpression of β-arrestin-2 did not influence *BiP* mRNA expression (Figure 6E), even though it had significantly downregulated BiP protein expression (Figure 4G). This inconsistency suggested that β-arrestin-2 might downregulate BiP by promoting its post-translational modification and subsequent degradation. β-arrestins are known to induce protein degradation through ubiquitination, and BiP has been reported to be downregulated by ubiquitination [11, 20]. Thus, we examined the ubiquitination of BiP in LPS-stimulated cells that ectopically expressed β-arrestin-2. We found that β-arrestin-2 increased the ubiquitination of BiP in a dose-dependent manner (Figure 6F). Furthermore, the ubiquitin-proteasome inhibitor MG132 increased BiP protein levels in β-arrestin-2-overexpressing cells, suggesting that the ubiquitin-proteasome pathway was involved in the degradation of BiP (Figure 6G). These results suggested that β-arrestin-2 may bind to BiP and promote its degradation via ubiquitination.

### Suppression of BiP by β-arrestin-2 leads to the release of BIK

**Figure 7:**
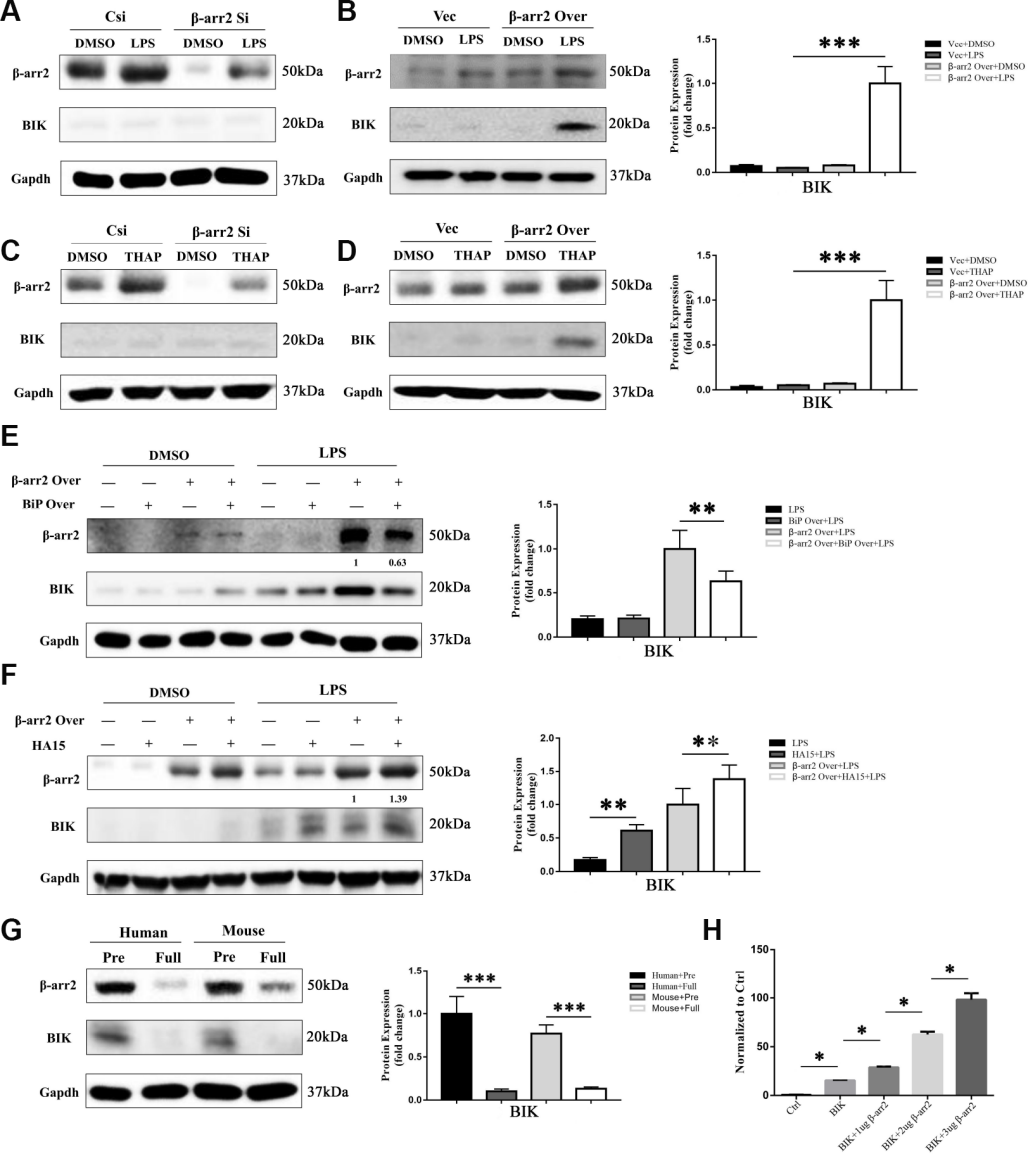
**Suppression of BiP by β-arrestin-2 leads to the release of BIK.** (**A**) Silencing of β-arrestin-2 had no effect on BIK levels in DMSO- or LPS-treated cells. (**B**) Overexpression of β-arrestin-2 increased BIK levels in LPS-stimulated cells, but not in DMSO-treated cells. (**C**) Silencing of β-arrestin-2 had no effect on BIK levels in thapsigargin (THAP)-stimulated cells. (**D**) Overexpression of β-arrestin-2 increased BIK levels in THAP-stimulated cells. (**E**) Co-expression of BiP attenuated BIK release in β-arrestin-2- overexpressing LPS-stimulated cells. (**F**) HA15 further upregulated BIK in β-arrestin-2-overexpressing LPS-stimulated cells. (**G**) Overexpression of β-arrestin-2 was associated with high levels of BIK in premature mice and humans. (**H**) β-arrestin-2 activated a *BIK* luciferase reporter (P<0.05).

BIK, a pro-apoptotic protein, has been reported to selectively form a complex with BiP in the tumor microenvironment [9, 10]. Thus, to explore the mechanism by which the downregulation of BiP induced apoptosis, we evaluated BIK expression in IECs. BIK expression was almost undetectable in the DMSO, LPS and thapsigargin control groups (Figure 7A–7D), and was not influenced by the knockdown of β-arrestin-2 (Figure 7A, 7C); however, BIK expression increased significantly when β-arrestin-2 was overexpressed in IEC-6 cells pretreated with LPS or thapsigargin (Figure 7B, 7D). Co-expression of BiP partially attenuated BIK release when β-arrestin-2 was overexpressed in LPS-stimulated cells (Figure 7E). On the other hand, when LPS-stimulated IEC-6 cells were treated with HA15, BIK was further upregulated after β-arrestin-2 induction (Figure 7F).

One interpretation of the above findings is that basal BiP levels are sufficient to sequester BIK under normal conditions and ER-stress conditions. However, β-arrestin-2 overexpression markedly reduces BiP levels, thus weakening the binding between BiP and BIK, ultimately promoting BIK release. This could explain why premature infants are prone to NEC, while full-term infants are relatively safe: because premature infants overexpress β-arrestin-2 and thus release excessive amounts of BIK. Western blotting of β-arrestin-2 and BIK expression in preterm and full-term human and mouse intestines confirmed this interpretation (Figure 7G).

Lastly, we conducted a luciferase reporter gene activation assay, and found that the *Bik* reporter was activated by β-arrestin-2 overexpression (Figure 7H). These results suggested that β-arrestin-2 induces NEC by promoting ER stress/BIK apoptotic signaling.

## DISCUSSION

**Figure 8:**
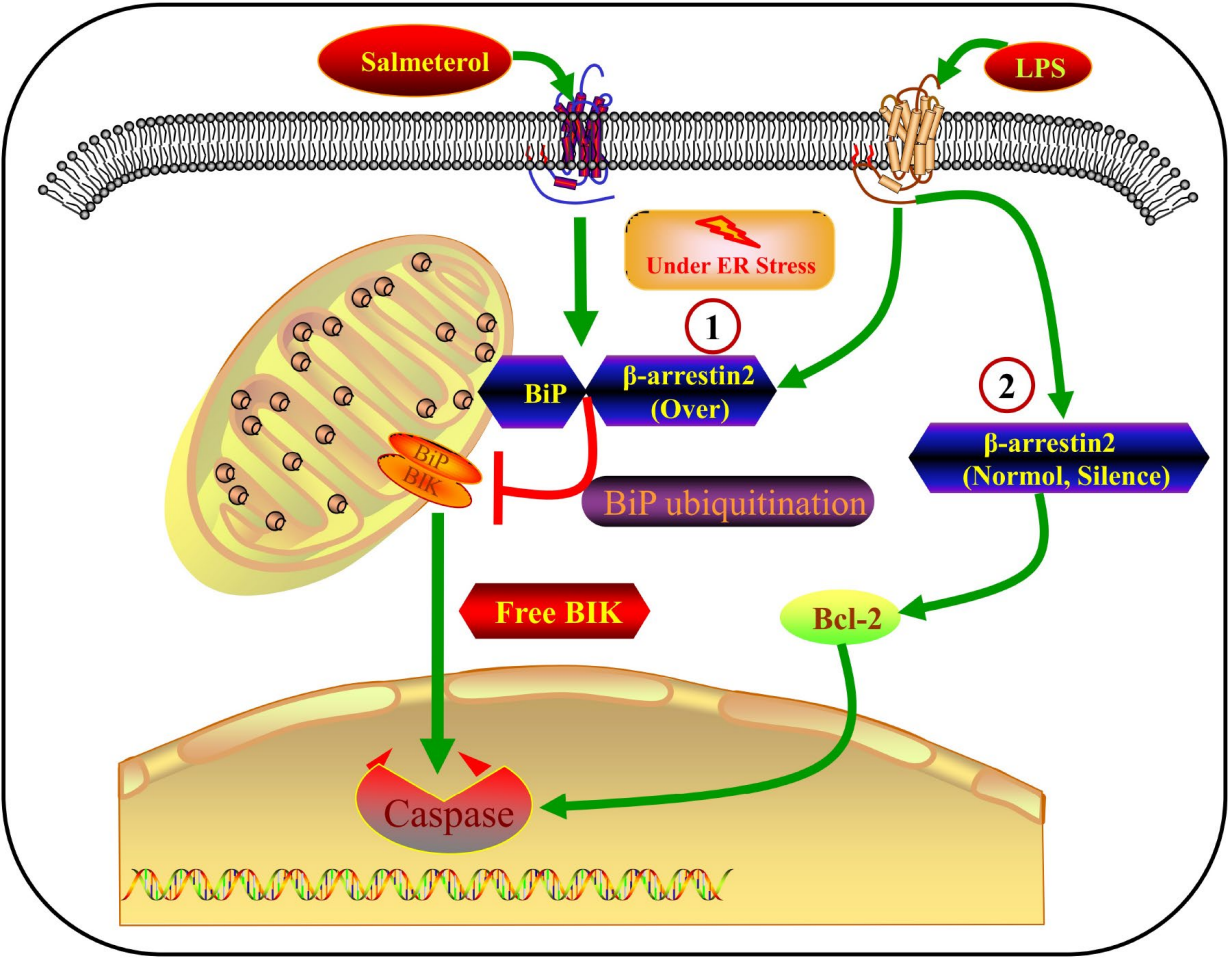
**Crosstalk between β-arrestin-2 and ER-stress-induced apoptosis.** LPS induces β-arrestin-2 expression. β-arrestin-2 binds to BiP (an effect enhanced by salmeterol) and inhibits its activity by promoting its ubiquitination. By suppressing BiP, β-arrestin-2 overexpression impairs the binding between BiP and BIK, thus promoting BIK release and caspase cleavage. When β-arrestin-2 expression is normal or silenced, LPS does not alter BIK expression, so other BCL-2-family proteins may promote apoptosis under such conditions.

In this study, we have presented a large body of evidence that β-arrestin-2 inhibits BiP in IECs, thus promoting the release of BIK and stimulating ER-stress-induced apoptosis. The major findings of this study are the following: 1) β-arrestin-2 was upregulated in human infants with NEC, in mouse and cell models of NEC, and in preterm humans and mice; 2) LPS or thapsigargin treatment upregulated β-arrestin-2, induced ER stress and promoted apoptosis in IECs; 3) silencing of β-arrestin-2 alleviated ER stress and apoptosis in NEC mice and LPS- or thapsigargin-treated IECs; 4) overexpression of β-arrestin-2 in LPS- or thapsigargin-treated IECs markedly increased ER stress and apoptosis; 5) β-arrestin-2 bound to BiP (an effect enhanced by salmeterol) and promoted its ubiquitination upon LPS stimulation, thus inhibiting BiP activity; 6) the BiP inhibitor HA15 promoted apoptotic marker expression in LPS-treated IECs and enhanced NEC development in mice; and 7) by suppressing BiP, β-arrestin-2 overexpression in LPS- or thapsigargin-treated cells may have impaired the binding between BiP and BIK, thus promoting BIK release; however, BIK levels were not affected by LPS or thapsigargin when β-arrestin-2 expression was normal or silenced, suggesting that other BCL-2-family proteins promote apoptosis under those conditions (Figure 8). All these results indicated that β-arrestin-2 promotes ER-stress-induced cell death by inhibiting BiP.

Although β-arrestin-2 was originally discovered as an attenuator of G-protein signaling, recent studies have demonstrated that it regulates signaling downstream of not only seven-transmembrane receptors, but also receptor protein tyrosine kinases, cytokine receptors and ion channel receptors [21–23]. The involvement of β-arrestin-2 in intestinal inflammation has remained ambiguous due to context-specific differences [24, 25]. For instance, Sharma et al. demonstrated that the absence of β-arrestin-2 in a DSS-induced colitis model increased the extent of mucosal inflammation and exacerbated the disease [24]. However, another report indicated that high β-arrestin-2 levels made mice more vulnerable to DSS-induced colitis [25]. Fan et al. observed that “caecal ligation and puncture”-induced caecum injury was more severe in β-arrestin-2 knockout mice than in wild-type mice [22]. However, there was no significant difference in liver damage between the knockout and wild-type mice, further demonstrating the context-specific activity of β-arrestin-2. Intriguingly, Chen et al. reported that deletion of β-arrestin-2 alleviated DSS-induced colitis during the acute stage, but was also associated with delayed mucosal repair of experimental colitis [26].

β-arrestin-2-dependent signaling has exhibited paradoxical characteristics not only in inflammation, but also in the regulation of apoptosis [27–29]. For example, under certain conditions, the β-arrestin-2-Src complex has been reported to transactivate the epidermal growth factor receptor (EGFR) tyrosine kinase pathway, thus inhibiting apoptosis and promoting proliferation by stimulating EGFR-dependent ERK1/2 activity [30]. In contrast, β-arrestin-2 has also been found to facilitate the direct scaffolding of ERK1/2, thereby enhancing the retention of active ERK1/2 in the cytosol and promoting apoptosis [31–33]. In general, β-arrestin-2 is involved in diverse signaling pathways that have not been completely delineated. In our study, we demonstrated for the first time that β-arrestin-2 promotes apoptosis in NEC. Although our findings may contradict certain previous reports describing anti-apoptotic effects of β-arrestin-2, we uncovered the specific biochemical pathway responsible for β-arrestin-2-enhanced NEC progression.

The proper ER stress response in the intestinal epithelium has been thought to be a protective strategy that maintains homeostasis [5]. However, intestinal secretory cells are particularly susceptible to ER stress [3]. Afrazi et al. found that BiP levels were higher in preterm and NEC intestinal tissues than in healthy control tissues from humans and mice [34]. The present study also indicated that the level of ER stress was higher in premature than in full-term intestines. NEC mice also exhibited higher ER stress levels than their corresponding controls. ER stress correlated well with β-arrestin-2 expression. *In vitro*, ectopic expression of β-arrestin-2 triggered ER stress and apoptosis upon LPS or thapsigargin stimulation, while silencing of β-arrestin-2 attenuated these effects. These data suggested that β-arrestin-2 promotes ER stress in NEC, which has not been reported previously.

As β-arrestin-2 is a multifunctional molecule, its binding partners have been progressively identified by various techniques, including co-immunoprecipitation and yeast two-hybrid screens [35]. Here, we found that β-arrestin-2 bound to and colocalized with BiP in co-immunoprecipitation and immunofluorescence analyses in IEC-6 cells. β-arrestin-2 inhibited BiP by binding to it, thus providing a theoretical explanation for the pro-apoptotic effects of β-arrestin-2. Pretreatment with HA15 exacerbated the pro-apoptotic effects of LPS treatment, regardless of β-arrestin-2 expression (normal or overexpressed), implying that BiP is an important component of β-arrestin-2-induced ER stress and apoptosis. It is well known that BiP expression increases during ER stress, but little has been reported about the proteins that regulate BiP. Thus, our findings have extended our understanding of BiP by demonstrating that it can be downregulated by β-arrestin-2.

BiP has mainly been characterized for its anti-apoptotic effects in the tumor microenvironment [9]. In this study, we indirectly demonstrated that BiP also promotes survival in the intestinal inflammatory environment. For many solid tumors, hypoxia occurs in the microenvironment due to an insufficient or heterogeneous blood supply [36]. In mice, hypoxia is the most important inducer of NEC. Thus, it is possible that BiP exerts its anti-apoptotic activity in low-oxygen environments.

We also found that salmeterol enhanced the association of β-arrestin-2 with BiP, and even exhibited pro-apoptotic activity upon LPS stimulation. However, in a previous study, salmeterol exerted anti-inflammatory effects by preventing the activation of the NLRP3 inflammasome in LPS-stimulated primary bone marrow macrophages [37]. Thus, it is possible that β-arrestin-2 partners with both anti-apoptotic proteins like BiP and pro-inflammatory proteins like NLRP3 under the same stimuli. Different protein partners in different cell types will undoubtedly have different consequences, depending on the pathways involved. In our study, salmeterol may also have induced cyclic adenosine monophosphate (cAMP), the typical intracellular second messenger of β2ARs. Previously, the cAMP/protein kinase A (PKA) pathway was reported to promote apoptosis in NEC [38]. Whether both G-protein-biased (β2ARs/cAMP/PKA) and β-arrestin-2-biased (β2ARs/β-arrestin-2/BiP) signaling pathways induce apoptosis may be worthy of future study. As for the clinical application, it remains to be explored whether β2AR antagonists are effective treatments for NEC.

BIK activation can induce cell death by BAX-dependent or -independent pathways [39], and numerous studies have demonstrated that BiP and BIK promote apoptosis in various cell types and diseases. The formation of the BiP/BIK complex at the ER prevents BIK from activating BAX and other pro-apoptotic factors in the BCL-2 family, including BAK, BAD, BID and BIM [10]. We found that ectopic β-arrestin-2 expression promoted the ubiquitination and degradation of BiP in LPS-treated cells, resulting in the release of BIK. However, LPS or thapsigargin treatment did not influence BIK expression when β-arrestin-2 expression was silenced or normal. Thus, other BCL-2-family proteins probably promote apoptosis when cytoplasmic β-arrestin-2 levels are normal or low.

There were some limitations to our study. The mice used in our research were whole-body knockouts, not intestine-conditional knockouts; thus, the function of β-arrestin-2 in NEC should be confirmed in intestine-specific β-arrestin-2 knockout mice. As β-arrestin-2 is highly expressed in the spleen, it may induce NEC not only through ER stress, but also through immunomodulatory pathways. These possibilities will be the subjects of ongoing studies.

In summary, we have identified a new signaling pathway in which increased binding between β-arrestin-2 and BiP promotes ER-stress-induced apoptotic signaling in NEC. As arrestin-related signaling pathways are currently being investigated as novel drug targets [40], β-arrestin-2 may not only have predictive value, but also provide a new therapeutic target in NEC.

## MATERIALS AND METHODS

### Reagents and antibodies

Primary antibodies for β-arrestin-2 (C16D9), CHOP (L63F7), and CC3 (Asp175) were purchased from Cell Signaling Technology; that for β2AR (bs-0947R) was purchased from Bioss; that for BiP (ab21685) was purchased from Abcam; and that for BIK (NB100) was purchased from Novus. LPS (O55:B5), HA15 (10 μM for 6 hours for cells; 10 mg/kg for mice) and thapsigargin (0.5 μM for 6 hours) were purchased from Sigma-Aldrich. MG132 (474790-1MG; 20 μM for 6 hours) was obtained from Calbiochem. Salmeterol (purity > 99%) was procured from Melone Pharmaceutical Co., Ltd (Dalian Digital and DNA Port, China). β-arrestin-2 and BiP siRNA were purchased from Qiagen. The BiP and β2AR plasmids and BIK reporter gene were synthesized by Hanbio Biotechnology Co, Ltd.

### Tissue samples

Frozen specimens from patients with NEC were obtained from the Department of General Surgery, Children’s Hospital of Shanghai. The acquisition of the tissue samples was approved by the Institutional Review Board at Children’s Hospital of Shanghai. We obtained written informed consent from all patients before including them in the study.

### Animals

C57BL/6J mice (five to seven days old) weighing 4-5 g were purchased from Shanghai Jiesijie Experimental Animal Co., Ltd. β-arrestin-2 knockout mice were obtained from the laboratory of Gang Pei. Mice were bred and maintained in the Animal Resource Centre, Chinese Academy of Sciences. Newborn premature mice were collected by Cesarean section on day E15 or E18 following isoflurane anesthesia. We used the NEC model described by Caplan et al. [41]. All animal experiments were approved by the Institutional Review Board at Children’s Hospital of Shanghai.

### Cell culture

The rat intestinal crypt cell line IEC-6 was purchased from the Cell Resource Center of the Chinese Academy of Sciences (Shanghai, China). The cells were cultured in Dulbecco’s modified Eagle’s medium supplemented with 10% fetal bovine serum and 1% penicillin/streptomycin at 37°C in a humidified 5% CO2 atmosphere. All cell experiments involving LPS treatment were conducted with 50 μg/mL LPS for 6 hours, as these were reported to be the optimum conditions for inducing NEC in IEC-6 cells [34].

### NEC evaluation

Immediately after the mice were sacrificed, terminal ileal samples were harvested and visually evaluated for typical signs of NEC. Shortly thereafter, the tissue specimens were fixed in 4% formalin for 24 hours, embedded in paraffin and stained with hematoxylin and eosin. We then histologically evaluated the specimens for the presence and/or degree of NEC using a previously published standard histologic scoring system (17208569, 7855004). Histologic changes in the intestines were graded as follows: grade 0, normal, no damage; grade 1, epithelial cell lifting or separation; grade 2, sloughing of epithelial cells to the mid-villus level; grade 3, necrosis of the entire villus; grade 4, transmural necrosis. Tissues with scores ≥2 were identified as NEC-positive. The remainder of the proximal ileum was frozen in liquid nitrogen and transferred to a −80°C freezer until protein and RNA analysis.

### IEC isolation

Epithelial cells were isolated from the mouse intestine as described by Zeineldin et al. [42].

### Immunostaining, real-time PCR and Western blot analysis

Human and mouse intestines were immunostained as previously described [43]. TRIzol^TM^ reagent (Invitrogen, #15596026) was used to extract RNA from both cells and tissue specimens, and cDNA was synthesized with PrimeScript^TM^ RT Master Mix (Takara, #RR036A). FastStart Essential DNA Green Master (Roche Diagnostics, #06924204001) was used to measure target gene amplification. Primers were synthesized by Sangon Biotech Co., Ltd, and the sequences are listed in Supplementary Table 2. Western blotting was also performed as previously described [43].

### Flow cytometry analysis

A fluorescein isothiocyanate (FITC) Annexin V Apoptosis Detection Kit with PI (BioLegend, #640914) was used to detect apoptosis. Cells were washed twice with cold BioLegend Cell Staining Buffer, and were resuspended in Annexin V Binding Buffer at a concentration of 0.25-1.0^10^ cells/mL. Then, 100 μL of this cell suspension was transferred to a 5-mL test tube. Next, 5 μL of FITC Annexin V and 10 μL of the PI solution were added. The cells were gently vortexed and incubated for 15 minutes at room temperature (25°C) in the dark. Subsequently, 400 μL of Annexin V Binding Buffer was added to each tube. The cells were analyzed with a BD FACSCanto II flow cytometer (BD Biosciences, San Jose, CA, USA).

### *BIK* luciferase reporter assay

IEC-6 cells were co-transfected with 1 μg of a *Bik* reporter plasmid and 1, 2 or 3 μg of *β-arrestin-2* cDNA cloned into a pcDNA3 expression vector containing Flag/HA-tag. Twenty-four hours post-transfection, cell extracts were prepared, and the relative luciferase activity was analyzed with a Dual-Light reporter gene assay system.

### Co-immunoprecipitation

Plasmids were co-transfected into IEC-6 cells, 1 μg per plasmid. Twenty-four hours post-transfection, cell extracts were prepared and incubated with Flag/HA beads for 12 hours. The extracts were then washed three times with phosphate-buffered saline Tween. Then, 100 μL of 1x loading buffer was added, and the samples were boiled for 10 minutes.

### Statistical analyses

All data are expressed as the mean ± standard deviation. Statistical analyses were performed with SPSS 24.0 software (SPSS, Chicago, IL, USA). Student’s t test and one-way analysis of variance were used to compare the groups. A difference was considered statistically significant when P was < 0.05.

## Supplementary Material

Supplementary Figures

Supplementary Tables
