# Supplementary material for: β-arrestin-2 enhances intestinal epithelial apoptosis in necrotizing enterocolitis
Source: Aging (Albany NY). 2019 Oct 14;11(19):8294–312. doi: 10.18632/aging.102320 (PMC6814604; doi:10.18632/aging.102320)
Supplement: Supplementary Figures [file aging-11-102320-s002.pdf]

SUPPLEMENTARY FIGURES

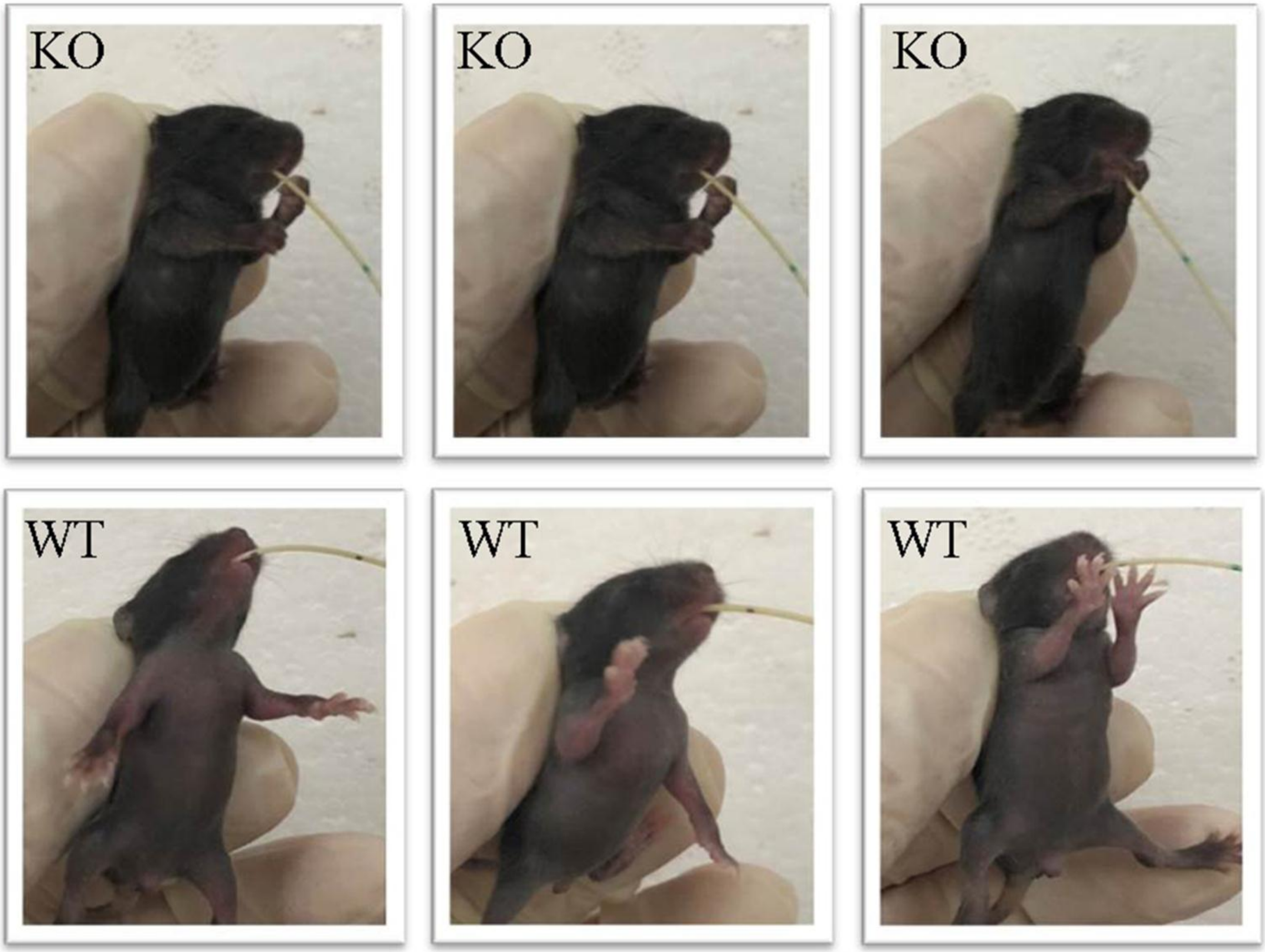

Supplementary Figure 1. When we started inserting the disposable silicone catheter (19Fr for newborns) into mice stomach, the KO mice behaved just like a hungry baby and hold the tube tightly with sucking action. However, the WT mice desperately struggled and rejected the tube.

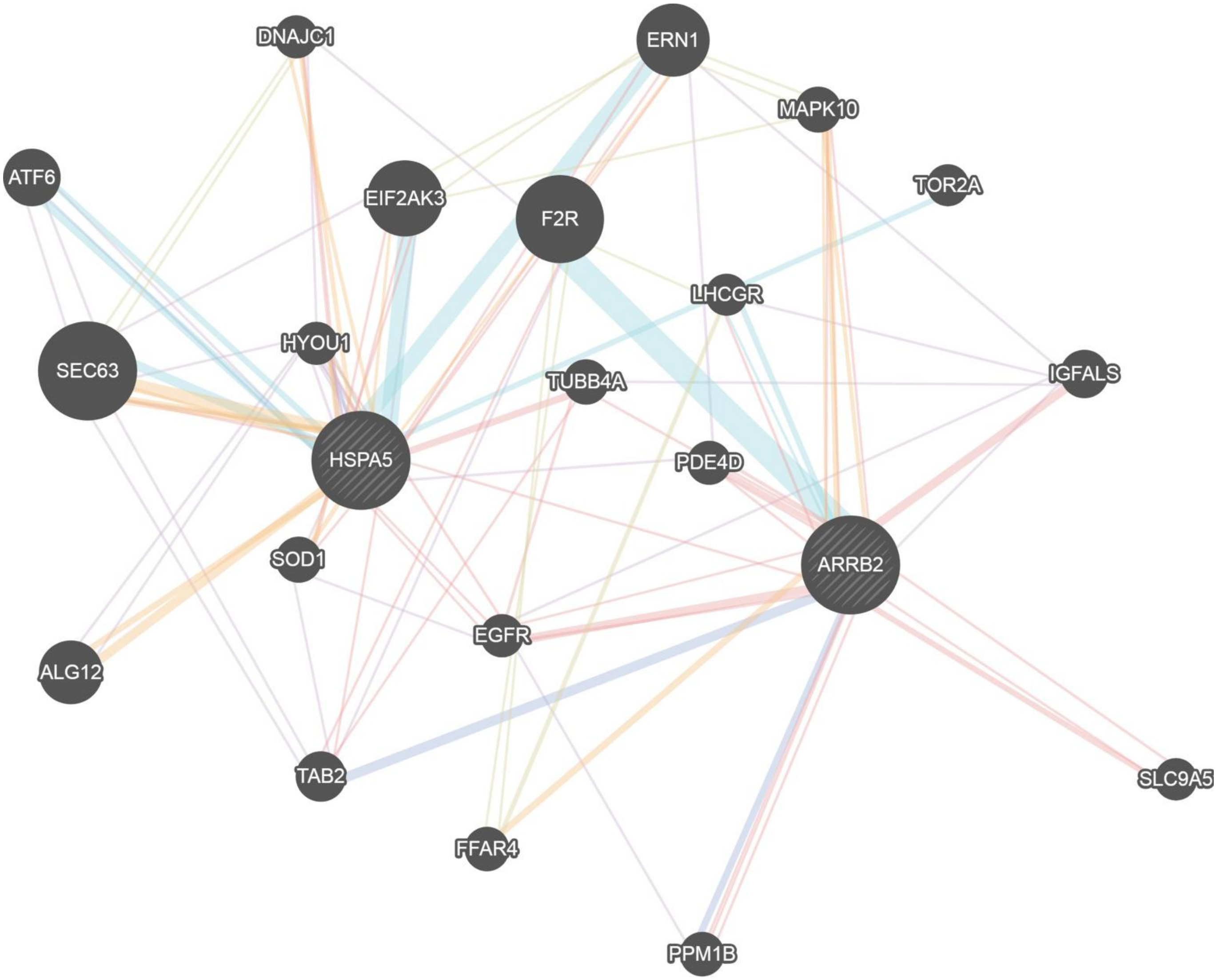

Supplementary Figure 2. Gene-Mania analysis showed an interaction between BiP and β-arrestin-2.
