# Supplementary material for: β-arrestin-2 enhances intestinal epithelial apoptosis in necrotizing enterocolitis
Source: Aging (Albany NY). 2019 Oct 14;11(19):8294–312. doi: 10.18632/aging.102320 (PMC6814604; doi:10.18632/aging.102320)
Supplement: Supplementary Tables [file aging-11-102320-s001.pdf]

SUPPLEMENTARY TABLES

Supplementary Table 1. Demographic and clinical characteristics of NEC patients and the control.

Patient code	Diagnosis	Gender	Gestational age at birth (weeks + days)	Postnatal age at surgery (days)	Birth weight (grams)
20113154	NEC	Male	33+5	84	1950
20088259	NEC	Male	35+4	8	2040
20073407	NEC	Male	29+6	19	1325
20063735	NEC	Male	31+5	43	1850
20054858	NEC	Female	35+4	83	1800
20066114	NEC	Male	30+2	33	1450
20021082	NEC	Female	31+6	14	1630
20043312	NEC	Female	26+3	89	910
20057380	NEC	Male	27+6	8	1350
20051851	NEC	Male	30+2	23	1600
20073398	Small intestinal atresia	Male	32+5	1	2050
20019558	Small intestinal atresia	Male	34+4	25	2240
20134076	Small intestinal atresia	Female	33+3	2	1650
20129198	Small intestinal atresia	Female	36+4	1	2960
20019518	Small intestinal atresia	Male	33+5	102	2400
20027346	Small intestinal atresia	Male	29+2	60	1610
20130173	Small intestinal atresia	Male	37	99	2760
20133280	Small intestinal atresia	Male	39+6	3	3580
20037411	Small intestinal atresia	Female	39+2	6	3140
20077303	Small intestinal atresia	Female	39+4	1	3370

Supplementary Table 2. Sequences of oligonucleotide primers.

Target gene	Primer sequences (5′ to 3′)
β-arrestin2	(F) GTCTTCAAGAAGTCGAGCCCT
(mouse)	(R) CACGAACACTTTCCGGTCCT
β-arrestin2	(F) AGCACCGCGCAGTACAAGT
(rat)	(R) CACGCTTCTCTC GGTTGTCA
β-arrestin2	(F) GTCGAGCCCTAACTGCAAG
(human)	(R) ACAAACACTTTGCGGTCCTTC
BiP	(F) CCAAGAGAGGGTTCTTGAATCTCG
(Rat)	(R) ATGGGCCAGCCTGGATATACAACA
β2AR	(F) GGTTATCGTAATGGCCATCGTGTTTG
(rat)	(R)TGGTTCGTGAAGAAGTCACAGCAAGTCTC
